# Multi-Round Trust Game Quantifies Inter-Individual Differences in Social Exchange from Adolescence to Adulthood

**DOI:** 10.5334/cpsy.65

**Published:** 2021-10-14

**Authors:** Andreas Hula, Michael Moutoussis, Geert-Jan Will, Danae Kokorikou, Andrea M. Reiter, Gabriel Ziegler, NSPN Consortium, Ed Bullmore, Peter B. Jones, Ian Goodyer, Peter Fonagy, P. Read Montague, Raymond J. Dolan

**Affiliations:** 1 Austrian Insitute of Technology, Vienna, Austria; 2 Wellcome Trust Centre for Neuroimaging, University College London, London, United Kingdom; 3 Institute of Psychology, Leiden University, Leiden, the Netherlands; 4 Anna Freud Centre, London, United Kingdom; 5 Lifespan Developmental Neuroscience, Faculty of Psychology, Technische Universität Dresden, Germany; 6 Department of Neurology, Max-Planck-Institute for Human Cognitive and Brain Sciences, Leipzig, Germany; 7 Centre for Cognitive Neurology and Dementia Research, Magdeburg, Germany; 8 German Center for Neurodegenerative Diseases, Magdeburg, Germany; 9 NSPN Consortium, UK; 10 Department of Psychiatry, University of Cambridge, Cambridge, United Kingdom; 11 Cambridgeshire and Peterborough National Health Service Foundation Trust, Cambridge, United Kingdom; 12 Medical Research Council/Wellcome Trust Behavioural and Clinical Neuroscience Institute, University of Cambridge, Cambridge, United Kingdom; 13 Max Planck University College London Centre for Computational Psychiatry, London, United Kingdom; 14 Research Department of Clinical, Educational and Health Psychology, University College London, United Kingdom; 15 Human Neuroimaging Laboratory, Virginia Tech Carilion Research Institute, Roanoke, Virginia, United States of America; 16 Department of Physics, Virginia Polytechnic Institute and State University, Blacksburg, Virginia, United States of America

**Keywords:** Development, Trust, I-POMDP, Gender Difference, IQ Effects, Model Based, Age, Adolescent, Risk Aversion, Socio-Economic Status

## Abstract

Investing in strangers in a socio-economic exchange is risky, as we may be uncertain whether they will reciprocate. Nevertheless, the potential rewards for cooperating can be great. Here, we used a cross sectional sample (n = 784) to study how the challenges of cooperation versus defection are negotiated across an important period of the lifespan: from adolescence to young adulthood (ages 14 to 25). We quantified social behaviour using a multi round investor-trustee task, phenotyping individuals using a validated model whose parameters characterise patterns of real exchange and constitute latent social characteristics. We found highly significant differences in investment behaviour according to age, sex, socio-economic status and IQ. Consistent with the literature, we showed an overall trend towards higher trust from adolescence to young adulthood but, in a novel finding, we characterized key cognitive mechanisms explaining this, especially regarding socio-economic risk aversion. Males showed lower risk-aversion, associated with greater investments. We also found that inequality aversion was higher in females and, in a novel relation, that socio-economic deprivation was associated with more risk averse play.

## Introduction

Socio-economic interactions with strangers are inherently risky. However, despite knowing little about potential partners’ intentions or probity, we may nevertheless choose to trust them to achieve greater gains for ourselves and to satisfy our own tastes for equity. Learning how much to trust strangers is particularly important during adolescence, as the community of people with whom we interact expands rapidly beyond the bounds of family and local familiarity, and because exploratory collaboration forms a spring-board for longer-term relationships (see Crone & Dahl, 2012, Nelson et al, 2005). Here, we quantify the development of the willingness to trust strangers across age 14 to 25.

Economic games offer a rigorous tool for examining the financial, risk-benefit trade-off associated with trusting others. Trust games of various kinds have been utilized to study social investment behaviour/trust in diverse samples of adolescents (see van den Bos et al, 2010, van den Bos et al, 2011, van den Bos et al, 2012, Belli, Rogers & Lau, 2012, van de Groep 2018) and even young children (see Rosati et al, 2019), with findings varying considerably. Some of these studies have suggested that trust behaviour (investing in an unknown partner with hope of reciprocity) is modulated by the amounts endowed to the investor before reciprocation (which determines ‘socio-economic exchange risk’) and by sex (female adolescents tending on average to invest less in unknown others). However, results have not been consistently replicated. Measuring the willingness to invest in anonymous partners during adolescence and young adulthood has yielded conflicting results (for increases see Sutter & Kocher, 2007; van den Bos et al, 2010, for decreases see Derks, Lee & Krabbendam, 2014, with an overview in van de Groep, 2018). Findings likely depend on the concrete choice of paradigm and age bracket.

A standard work-horse in the study of psychiatric disorders is a multi-round variant of the ‘investor-trustee’, or multi-round trust task (McCabe, Rigdon & Smith, 2003, King-Casas et al, 2005) (called the MRT). In the task, one player acts as ‘investor’, and the other as a ‘trustee’. On each of 10 rounds of the game, the investor receives a regular ‘wage’ from the experimenter and decides on how much of it to invest into the trustee. The experimenter triples the investment, before the trustee chooses the amount of money to return to the investor. Thus, if the investor takes a risk by investing in the trustee, as the amount of repayment is uncertain, but if the trustee indeed pays back an appropriate amount, both parties benefit.

The mutual dependency between investor and trustee for maximising their respective outcomes permits a fine-grained study of the ability to establish and maintain cooperation. This task has thus been used to understand the neural underpinnings of personality disorders, the social deficits found in autism, and also aspects of other disorders (see King-Casas et al, 2008, Koshelev et al, 2010, Mellick, Sharp, & Ernst, 2019).

In recent research, detailed computational models of this task (see Ray et al, 2008, Xiang et al, 2012, Hula, Montague & Dayan, 2015, Hula et al, 2018) were built. These capture the dynamics of the exchange using a few parameters that quantify key characteristics of each participant. These computational models describe behaviour as arising from an interactive process, wherein participants cannot directly observe the characteristics of others, but can gather information about them from the exchange. Participants are parameterised by their social preference factors (i.e. a socio-economic exchange risk and inequity aversion), their capacity to create and avoid ruptures in cooperation, their prior beliefs about these characteristics of their partners, and the sophistication of their theories of other people’s minds, all of which we will describe in detail below. In previous work, risk aversion has turned out to be particularly important in describing investors; as this captures a propensity to prefer money that is not put at risk through investing with the trustee over money that is returned through the interaction. Notably, risk-taking is widely discussed to change markedly from adolescence to adulthood (see Steinberg, 2004, Romer, Reyna, & Satterthwaite, 2017, Defoe et al, 2015), but also to be dependent on other individual factors like sex (see Bach et al, 2020, Reniers et al, 2016, Byrnes, Miller & Schafer, 1999, van Duijvenvoorde et al, 2016), socio-economic status and IQ.

Here, we leverage a large (n = 784) cross-sectional sample of adolescents and young adults to investigate the relationship of behaviour in the MRT with individual differences regarding age, IQ, sex and socio-economic status. To gain detailed insight into this question, we use our novel computational model to dissociate latent factors contributing to decision-making in this task, in order to link them to our inter-individual variables of interest (age, IQ, sex and socio-economic status). Following several examples in the literature, which point towards changes in trust and reciprocity during adolescence (see Fett et al, 2014, Eisenberg et al, 1991, Derks, Lee & Krabbendam, 2014, Belli, Rogers & Lau, 2012), we found that differences in risk aversion in the MRT statistically explained a substantial portion of the variability of the investment behaviour observed across participants. Regarding inter-individual differences, we found a marked trend towards higher investments and lower risk aversion with increasing age and IQ.

Regarding sex differences, male participants invested more than females. In addition, we found indications of a difference in the attitude towards outcome inequality between male and female subjects, with females being classified as more inequality averse.

Finally, we discovered a previously unreported effect of socio-economic status (SES) on risk-aversion in the MRT. There was a significant effect towards higher risk aversion for subjects living in more adverse socio-economic conditions. Together, these effects show that individual differences can have an important impact on the propensity to invest, and possibly to profit in real-life economic exchanges with strangers.

## Methods

### Subjects and Data Collection

We administered the MRT game to a large sample of young people from London or Cambridge who participated in the Neuroscience in Psychiatry Network study (NSPN). We also collected basic demographic variables (age and self-reported sex, referred to as sex), Socio-Economic status (Neighbourhood ‘households in poverty index’ of the Office of National Statistics 2014, a score that is higher for more adverse conditions) and the Wechsler Abbreviated Scale of Intelligence (WASI) Score (see Wechsler, 2008, Weiss et al, 2010) for IQ. We used raw IQ scores for our analysis. The sample was equally distributed between females and males and between the ages of 14 to 25. Participants were excluded if they currently received help for a psychiatric problem, if they had moderate or severe learning disability or suffered from a serious neurological disorder. For the main analysis, we included only participants for which the above mentioned 4 demographic variables (age, IQ, sex, SES) were fully available (n = 784 out of 788 participants who completed the multi round trust game, n = 403 female, age range 14–25, mean age 19.05, sd = 2.96).

A detailed description of the methodology of the NSPN study can be found in Kiddle et al, 2017.

Data and Code used can be found in a public *github repository*.

### Ethics Statement

Participants themselves, and, if they were younger than 16, their legal guardians, provided informed consent. All clinical investigation was conducted according to the principles expressed in the Declaration of Helsinki. The study was approved by the Cambridge Central Research Ethics Committee (12/EE/0250).

### The Multiround Investor – Trustee Task

Participants were instructed by trained research assistants about the veridical rules of the game. The task was administered as part of a larger battery of decision-making tasks (see Kiddle et al, 2017).

Participants played the role of the Investor. They were told that they would receive monetary rewards in proportion to their winnings, which was true. As a cover story, they were also led to believe that they were playing with a peer from the same study, playing anonymously from another site, who would also be paid in proportion to their own winnings. Importantly, they did not know the name, sex or any background of their partner. They were asked to play according to their own goals and preferences, rather than optimize an experimenter-specified goal. At the end of the whole study they were debriefed as to the true nature of their ‘partner’, which was a computer algorithm that emulated the behaviour of healthy adult Trustees (see King-Casas et al, 2005).

Participants were encouraged to play as best they could, but were told that the experimenter was interested in how young people made decisions during interactions according to their own preferences and values. Thus, unlike other tasks in the battery, they were not instructed to achieve any particular goal or outcome – this was up to them.

**Figure 1:**
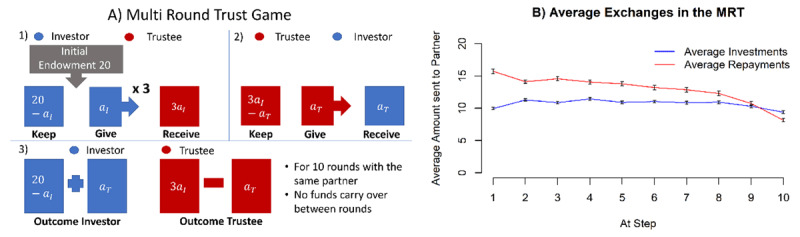
**A)** Schematic representation of the multi round trust game (MRT). **B)** Average Investments and Repayments in the MRT in this study. Errorbars are standard errors of the mean (SEM).

The rules of the game were as follows (see also ***Figure 1A***): In each of the ten rounds, the investor received an initial endowment of 20 monetary units, or play-coins, and could decide the amount (in whole coins) to transfer to the trustee. The experimenter trebled this quantity and then the trustee (in our case, the computer algorithm mentioned above) decided how many coins to return to the investor: this ranged between 0 coins and the total amount received. The repayment by the trustee was not increased by the experimenter. After the trustee’s action, the investor was informed of the outcome, and the next round started. We thoroughly tested the understanding of participants before the game started and encouraged them to ask any questions they had. All participants reported here understood the task well, agreed to play it and provided data of adequate quality for analysis.

### Model

We modelled behaviour using an interactive, partially observable Markov decision process (I-POMDP) (see Gmytrasiewicz & Doshi, 2005). We outline the model here, and describe the seven parameters 𝛳=(*α, ω, k, P, ζ, q*(*ζ*), *β*) that determine the behaviour of our modelled investors, with details on the employed internal state model, inference and statistical properties of the model to be found in Hula et al, 2018. In short, the I-POMDP is predicated on three structural characteristics: the rules of the task (which define an MDP); the assumed characteristics of the subjects playing the task; and the ignorance of the subjects about their partners (leading to partial observability), “partner” in this context always referring to the trustee. For computational reasons, we exploited prior studies to restrict the values of the parameters.

According to the I-POMDP characterization, the investor assumes that the trustee processes the information received through the task and makes choices in a structurally similar manner to the investor themselves. In our actual task, this is not formally true, since the trustee is simulated by a computer program that matches the current situation to a database of recorded interactions. Nevertheless, this assumption allows us to build a faithful model of the investors, and thereby interpret their individual characteristics.

We note that the parameters used here were discretized partly for computational reasons, because the interactive inference in the I-POMDP is quite costly (for *𝛼* and *ζ*). Furthermore, some of the parameters are discrete by nature (*k,P*). The remaining ones (*ω, q*(*ζ*), *β*) were discretized on a grid to allow for exhaustive search over the overall parameter space.

#### Inequality Aversion

We start with positive inequality aversion or guilt, *𝛼* ∈ {0, 0.4, 1}, the settings corresponding to no inequality aversion, slightly averse and fully averse (the grid for 𝛼 having been determined in Hula, Montague & Dayan, 2015). This quantifies how sensitive a subject is to advantageous unequal outcomes (see Fehr & Schmidt, 1999), i.e. how much they prefer an equal outcome. If χ_I_ denotes the current round outcome of the investor, and χ_T_ denotes the current round outcome of the trustee, then the subjective utility *u_I_* of the investor with inequality aversion *𝛼* is


1 \[
{u_I} = {\chi _I} - \alpha\ {\mathrm{max}}\left\{ {{\chi _I} - {\chi _T},{\mathrm{0}}} \right\}.
\]

where the outcome is


2 \[
{\chi _I} = \left( {{\mathrm{20}} - {a_I}} \right) + {a_T}.
\]

and


3 \[
{\chi _T} = {\mathrm{3}}{a_I} - {a_T}.
\]

That is, the utility for an investor with α > 0 is reduced if their associated trustee earns less than themselves. The equivalent is true for the trustee. For the investor, a critical contribution to the trustworthiness of the trustee can be framed as beliefs and learning of the trustee’s α.

If the trustee actually has a high value of α, then it is safe for the investor to make a substantial investment. However, in our anonymous setting, the investor does not know the trustee’s inequality aversion. Instead, the investor has to learn this from the trustee’s behaviour. In principle, the trustee would be in the same position relative to the investor’s inequality aversion. This makes the problem partially observable – at least some information is not known to the players, who therefore are assumed to perform approximate Bayesian inference to learn about these unknown factors.

#### Risk Aversion

The parameter ω ∈ {0.4, 0.6, 0.8, 1.0, 1.2, 1.4, 1.6, 1.8} quantifies the risk aversion of an investor, i.e. the relative value they accord to the (certain) amount that they keep and do not invest. This is quantified by modifying the notional outcome for the investor, *χ_I_*: If the amount the investor gives is denoted by *a_I_* and the trustee return is denoted by *a_T_*, then


4 \[
{\chi _I^{true}} = \omega \left( {{\mathrm{20}} - {a_I}} \right) + {a_T}.
\]

And


5 \[
{u_I^{true}} = {\chi _I^{true}} - \alpha\ {\mathrm{max}}\left\{ {{\chi _I^{true}} - {\chi _T},{\mathrm{0}}} \right\}.
\]

That is, the investor weighs the kept amount (20 – *a_I_*) more (ω > 1) or less (ω < 1) than the amount *a_T_* received through the interaction. This automatically weighs the variable (and hence uncertain) repayments differently from the certain amount that is kept. We assume that the investor believes her risk aversion to be “natural” in the sense that her model of the trustee will operate as if her actual degree of risk aversion was known. The trustee on the other hand is modelled as having a fixed belief of the investor’s risk aversion, dubbed *b_T_* (ω), which is fit in the same manner as the other parameters, i.e. through maximum likelihood estimation. Since all our subjects are investors, this parameter will not play a further role in the analysis.

#### Theory of Mind

The most conceptually complex parameter is the level of thinking (theory of mind (ToM) level) (see Costa-Gomes, Crawford & Broseta, 2001, and related Camerer, Ho & Chong, 2004) *k* ∈ {0, 1, 2, 3, 4}. Level k-thinking classifies beliefs about the nature of other players by the number of mentalizing steps they employ to model the interaction. Take inequality aversion. A level 0 investor (‘she’) would build a model of the trustee (‘he’) based on her beliefs about his degree of “inequality aversion” (see Ray et al, 2008, Xiang et al, 2012, Hula, Montague & Dayan, 2015, Hula et al, 2018)]. A level 1 investor would also model the trustee’s beliefs about her own degree of inequality aversion. Thus, the investor has to put herself into the trustee’s shoes to mentalize what he believes about her. This recurses – thus a level 2 investor would model the trustee’s beliefs about her own beliefs about his inequality aversion. In general if the investor is aware that “my trustee partner might think on a (maximum) level k-1” then the investor’s decision making will be at level k (since they take the level k-1 acting into account), however we make the further simplifying assumption that a level k investor considers her partner to operate *exclusively* at level *k* – 1. The alternative would be a level k investor potentially considering the trustee to operate at all levels below k, but at least at level k-1 with positive probability, which would however diffuse the choice preferences too much within the 10 round interaction of the MRT.

The same is true for the trustee modelling the investor. In our models, this potentially unbounded iterative structure of beliefs was limited to four steps, since higher levels do not appear to lead to notably different behaviours (see Hula et al, 2018).

#### Planning

Next comes parameter *P* ∈ {1, 2, 3, 4}, which quantifies the number of future exchanges to be taken into account in thinking forward. This is called the subject’s planning horizon and for the investor it has two main effects (detailed in Hula, Montague & Dayan, 2015): longer planning horizons allow for the execution of consistent gameplay strategies and allow to pre-empt exploitation by the partner by realizing initial cooperation might be succeeded by exploitation of the obtained reputation of trust worthiness (in particular taking the form of the trustee no longer repaying fair splits).

#### Irritability

The parameter *ζ* ∈ {0, 0.25, 0.5, 0.75, 1}, called irritability, provides a model of punishment actions, i.e. sudden investments or repayment close to 0, for instance after an action by the trustee that did not satisfy the expectations of an investor. We provide some more detail on this parameter, as it (and the following irritation awareness below) might be the most idiosyncratic of the model parameters, since it arose out of the study of Borderline Personality Disorder (BPD) data (see also King-Casas et al, 2008). This parameter governs a potential state shift to and from an irritated state, characterised by setting the planning horizon, *P*, to 0, removing any positive inequality aversion/guilt, *α*, and failing to model the mental state of the other person (i.e., when either person is acting irritated, their theory of mind level *k* is locally set to –1, which implies that they fail to mentalize about the other player at all). The values of all other parameters are kept equal to those in the “nonirritated” state. The size of the irritation parameter controls how quickly a subject switches from the non-irritated state to the fully irritated state i.e. after an irritation a subject with *ζ* = 0.25 will follow a mixture policy of 25% irritated action preference and 75% non-irritated action preference, while a subject with *ζ* = 0.75 will follow a 75% irritated action preference and 25% non-irritated action preference. Further irritation events increase the shift to the irritated policy i.e. a subject with *ζ* = 0.25 following a 25% irritated policy getting irritated immediately again will shift to a 50% irritated policy, while a subject with *ζ* = 0.75 following a 75% irritated policy will shift to a 100% irritated policy. Irritation can be undone by reciprocating or raising investment despite the irritated action and this shifts the policy making back from the irritated state at an analogue pace to the shift towards irritation. In this case the irritated state is left again and the “normal” method for making choices governs choice again (additional details in Hula et al, 2018).

#### Irritation Awareness

Additionally, subjects may be able to infer, whether their partner might be irritable. This is realized via approximate Bayesian inference on the partner’s irritability parameter based on 5 possible initial belief distributions represented in the parameter *q*(ζ) ∈ {0, 1, 2, 3, 4}, called “irritation awareness”. This parameter governs (in a monotonically increasing manner) how sensitive choice making is to the possibility of partner irritability (see Hula et al, 2018 for further details), i.e. a subject starts out with different prior weights on the 5 possible values of the partner’s ζ, which correspond to “never irritable”, “unlikely to be irritable”, “possibly irritable”, “likely irritable” and “certainly irritable”.

#### Interactive Inference

Two of the parameters (positive inequality aversion/guilt and irritability) are assumed to be inferred by the investors during the interaction, while the investor assumes all other parameters, (risk aversion, initial irritation belief and planning), to be the same in the partner as in themselves. We followed this strategy because computational constraints and data constraints (i.e., the number of rounds played per subject) limit the number of variables that we can model to be inferred jointly. Other candidates besides inequality aversion and irritability to be inferred interactively might be planning, theory of mind or risk aversion. However, for theory of mind and planning the amount of evidence gathered interactively during the ten rounds of the game (rather than from the concluded full interaction) would likely be little and considerable assumptions on the most likely level and planning horizon would have to be in place for the actor to benefit from this, which in turn is likely to yield again a situation as with the currently fixed parameters. For risk aversion, we did not consider this since there is currently no mechanism in the model that would shift risk aversion in one way or another and hence we did not expect it to yield interesting new forms of interaction. Future models will investigate this further.

We note that interactive inference by the investor was limited in two cases: If the investor invested 0 (and hence the trustee could not act) and if the investor chose the “invest 5” action: In this case the fair split is achieved by the trustee returning nothing and hence the trustee action does not carry information about inequality aversion preferences or irritability of the trustee.

#### Inverse Temperature and Choice Making

The investor model can calculate so-called action values *Q*(*a*|*h*). These quantify the expected value over the future planning horizon of executing action *a* (an investment or a return) given that the past history of investments and returns is *h*.

The action values determine the probability of choosing an action *a* through a logistic softmax function (Eq. 6) with a temperature parameter \[
\beta \in \left\{ {{\textstyle{1 \over 4}},{\textstyle{1 \over 3}},{\textstyle{1 \over 2}},{\textstyle{1 \over 1}}} \right\}
\] :


6 \[
\mathbb{P} \left[ {a{\mathrm{|}}h} \right] = \frac{{{e^{\beta Q(a|h)}}}}{{\mathop \sum \nolimits_c {e^{\beta Q(c|h)}}}}
\]

Again, for computational convenience, we constrain *a* for the investor to take one of five possible values, corresponding to {[0,2], [3,7], [8,12], [13,17], [18,20]}, being treated as investments of 0, 5, 10, 15 or 20 respectively. Similarly, the trustee returns are discretized to rounded fractions {0, 1/6, 1/3, 1/2 or 2/3) of the tripled investment, i.e., the amount they receive. Returns above 2/3 of the received amount were very rare.

#### Classification and Parameters

Each subject is classified according to the parameter vector which generated the lowest negative log likelihood for the observed interaction. That value was found by search over all possible parameter values.

A summary of all parameters and their ranges can be found in ***Table 1*** below.

**Table 1:** Model Parameters, Parameter Ranges and Interpretation of the parameters.


PARAMETER SYMBOL	PARAMETER NAME	RANGE	MEANING

***α***	Inequality Aversion/Guilt	{0, 0.4, 1}	Degree of sensitivity to an unfair outcome against the other player.

***ω*,b^T^ (*ω*)**	Risk Aversion	\[ \left\{ {\begin{array}{*{20}{c}} {0.4,\;0.6,\;0.8,\;1,\;}\\ {1.2,\;1.4,\;1.6,\;1.8} \end{array}} \right\} \]	Multiplier for value of money kept over money returned by the partner.

***k***	ToM Level	{0, 1, 2, 3, 4}	Number of recursive reasoning steps in representing beliefs of the other player.

***P***	Planning	{1, 2, 3, 4}	Number of steps ahead planned into the interaction.

***ζ***	Irritability	{0, 0.25, 0.5, 0.75, 1}	Measure of shift towards punishment behaviour, when experiencing below expectation partner actions.

**q(ζ)**	Irritation Awareness	{0, 1, 2, 3, 4}	Awareness of partner irritability. 0 = unaware, 4 = partner for sure irritable.

***β***	Inverse Temperature	\[ \left\{ {\frac{1}{4},\frac{1}{3},\frac{1}{2},\frac{1}{1}} \right\} \]	Measure of stochasticity in choices given their expected utilities.


#### Investment Structure

The average investment and repayment values per round are shown in ***Figure 1B***. If the investor gives 10, then any return from the trustee of more than 10 nets the investor with as much as, or more than the trustee, and so represents reliable or over-reciprocating cooperation. ***Figure 1B*** shows that the trustee starts out in this manner (automatically reflecting the characteristics of the human participants on which its choices are based); this might coax the investor into investing more. It also reveals that the average investment remained remarkably stable. The actual individual investment trajectories are related to the model mechanisms in more complex ways, such as decreasing investments towards the end of the interaction (an effect of planning, see Hula, Montague & Dayan, 2015) and substantial drop offs of investments during the interaction (for irritable investors, see Hula et al, 2018).

#### Risk Aversion Confusion Analysis

**Figure 2:**
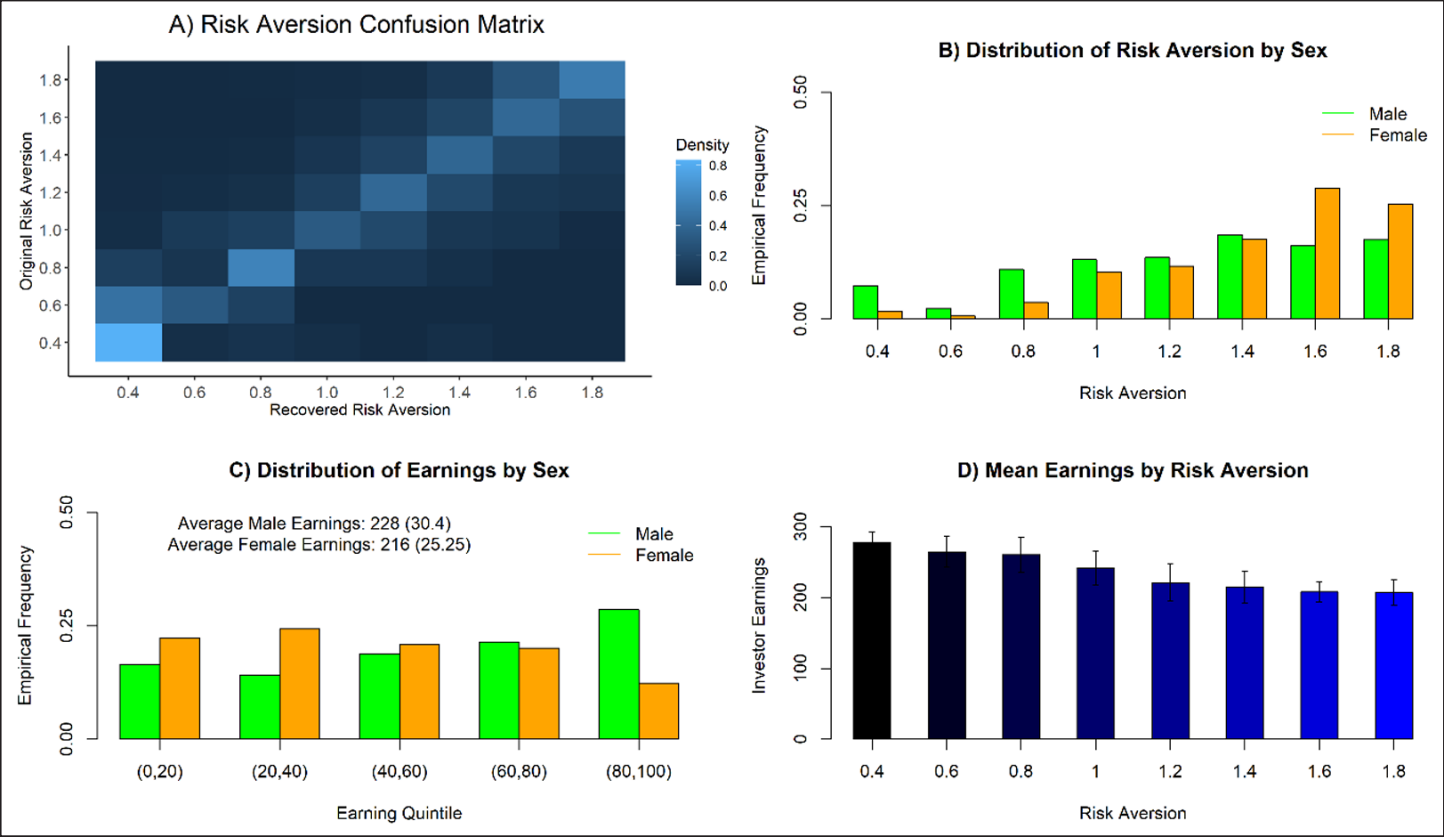
From top left to bottom right. **A)** Confusion matrix: trajectories were sampled from the generative model using the full collection of parameters inferred from each subject (y-axis); then new parameter values were re-estimated from these trajectories (x-axis). The matrix shows the conditional probability of the re-estimated value of risk aversion as a function of the generating risk aversion. Lighter values indicate higher probabilities. Rows sum to 1. **B)** Frequency of occurrence of each risk aversion value, separated by sex. **C)** Average earnings separated by sex and classified according to membership in total earnings quintiles in this study. **D)** Average investment levels separated by subjects’ Risk Aversion parameter. Error bars are standard deviations (SD).

To confirm that our generative model pin-pointed key parameters for individual participants, a prerequisite for analysis of individual variation, we used the model to create sample trajectories (1 for each dyad) based on the parameter values that we inferred from the human subjects. Using these generated dyads, we estimated a full set of new parameters. ***Figure 2A*** shows the confusion matrix for the parameter that turns out to be most critical: risk aversion. This matrix compares the actual value of the risk aversion on the basis of which a trajectory was created to the value of risk aversion that we inferred from the trajectory. This quantifies the quality of inference about the crucial risk aversion parameter. We found that parameter recovery of risk aversion was very stable (with misestimations consisting primarily of a shift to a directly adjacent parameter value, which would have limited impact on the kind of monotone relationships we are investigating below) and thus inferences based on the risk aversion parameter are justified.

#### Analysis Set-Up

The prime expression of trust in the MRT is the willingness to invest in a partner. Our first hypothesis thus was, that the parameter estimates would correlate with the average/sum investment levels. We thus proceeded to estimate the proportion of variance in the total investment *y* across all rounds that could be accounted for linearly (using the function “lm” implemented in R, see R Core Team, 2017) by the fitted parameters treated as discrete ordinal quantities (In Eq. 7 below, *ϵ* denotes an error term):


7 \[
y = b_{\mathrm{0}}^\vartheta + b_k^\vartheta k + b_P^\vartheta P + \;b_\alpha ^\vartheta \alpha + \;b_\omega ^\vartheta \omega + b_\beta ^\vartheta \beta + \;b_\zeta ^\vartheta \zeta + \;b_{q\left( \zeta \right)}^\vartheta q\left( \zeta \right) + \epsilon.
\]

This explained a full 69% of the variance (adjusted R^2^) of the total investment, while the socio-economic risk aversion parameter ω as a sole regressor can account for 49% (adjusted R^2^) of the variance. This is reasonable, as the sum investments would be most affected by a parameter governing the preference of money kept over money gained directly.

We then examined how demographic variables may account for social-cognitive characteristics, as captured by model parameters. To assess this, we used linear and quadratic regression for the metric parameters (risk aversion, temperature), and ordinal regression for the others (ToM level, planning, inequality aversion, irritability and the irritation belief). To make the effects of the demographic variables in the linear models more easily comparable, we standardized them by Z-Scoring (subtracting the respective mean and dividing by the standard deviation) before squaring them and before using them in the regressions.

Using *φ* as a placeholder for variable of interest for the metric variables, we considered models of the form


8 \[
\phi = b_{\mathrm{0}}^{\;\phi } + b_{Age}^{\;\phi }Age + b_{IQ}^{\;\phi }IQ + \;b_{SES}^{\;\phi }SES + \;b_{sex}^{\;\phi }sex + b_{Ag{e^{\mathrm{2}}}}^{\;\phi }Ag{e^{\mathrm{2}}} + b_{I{Q^{\mathrm{2}}}}^{\;\phi }I{Q^{\mathrm{2}}} + \;b_{SE{S^{\mathrm{2}}}}^{\;\phi }SE{S^{\mathrm{2}}} + \epsilon
\]

Here, for *sex*, we conventionally coded male as 0 and female as 1.

Using *φ* again as a placeholder for the variable of interest, for ordinal regressions (implemented in R (R Core Team, 2017) via the “polr” function), we studied


9 \[
l(\mathbb{P} [\phi < i]) = b_{{\mathrm{0}},i}^{\;\phi } + b_{Age}^{\;\phi }Age + b_{IQ}^{\;\phi }IQ + \;b_{SES}^{\;\phi }SES + \;b_{sex}^{\;\phi }sex + b_{Ag{e^{\mathrm{2}}}}^{\;\phi }Ag{e^{\mathrm{2}}} + b_{I{Q^{\mathrm{2}}}}^{\;\phi }I{Q^{\mathrm{2}}} + \;b_{SE{S^{\mathrm{2}}}}^{\;\phi }SE{S^{\mathrm{2}}} + \epsilon
\]

With a logit link function *l* and a given level i. We only report significance levels for the variables and not for the intercepts in the ordinal regression.

We started with these full models and employed stepwise model selection, based on the Akaike Information Criterion (AIC), in order to arrive at the most parsimonious model to describe our data. Model selection via the AIC was implemented using via the “stepAIC” function in the statistics language R (see (R Core Team, 2017)). At each step, this considers whether AIC improves upon removing just one term, so tests 7 models in the first step, 6 models in the second, etc., until the AIC can no longer be improved. Below we give the winning model for each parameter and discuss the significant and marginally significant (at p < 0.05 and p < 0.1 respectively) surviving variables in the winning model.

We illustrate the correlations between the demographic variables below in ***Table 2*** (Kendall’s tau coefficients) and ***Table 3*** (p-values).

**Table 2:** Kendall’s tau correlations (below diagonal) among the demographic variables employed in this study.


	*AGE*	*IQ*	*SES*	*SEX*

***Age***	1			

***IQ***	0.088	1		

***SES***	0.1	–0.046	1	

***Sex***	–0.004	–0.09	–0.047	1


**Table 3:** p-values (below diagonal) for the correlations among the demographic variables employed in this study. Asterisk (*) signifies correlations with a p-Value below 0.05.


	*AGE*	*IQ*	*SES*	*SEX*

***Age***	0			

***IQ***	0.00026*	0		

***SES***	0.000021*	0.056	0	

***Sex***	0.88	0.002*	0.11	0


## Results

The model captured trial-by-trial behaviour well, the negative loglikelihood (NLL) being 8.13, corresponding to an average 0.43 likelihood in the model for the actually made subject choice, based on the current game history and the model parameters (uniform chance = 0.2). In the following we present the outcomes of model selection on the regression models between demographic factors and model parameters.

### Relations between socioeconomic risk aversion and demographic variables

For risk aversion (*φ* = ω), the ultimate model was:


10 \[
\omega = b_{\mathrm{0}}^{\omega} + b_{Age}^{\omega}Age + b_{IQ}^{\omega }IQ + \;b_{SES}^\omega SES + \;b_{sex}^\omega sex + \;b_{SE{S^{\mathrm{2}}}}^\omega SE{S^{\mathrm{2}}} + \epsilon,
\]

We found highly significant negative relationship between Risk Aversion and Age and raw (non-age-standardized) IQ, and a significant relationship with sex (respectively: \[
b_{Age}^\omega = - 0.3
\] , *p* = 10^–5^ for Age, \[
b_{IQ}^\omega = - 0.43
\] , *p* = –10 for IQ and \[
b_{Sex}^\omega = 0.82
\] , *p* = 10^–10^ for sex) as well as a positive relation to the SES score \[
b_{SES}^\omega = 0.19
\] , *p* = 0.04) and a negative relation to the squared SES score \[
b_{SE{S^2}}^{\omega} = - 0.12
\] , *p* = 0.01).

We investigated the effects on risk aversion in more depth, as risk aversion explained a high proportion of the variance in total investments and was strongly correlated with the socio-demographic variables. We plot the distribution of the risk aversion parameter split by sex in ***Figure 2B***. Every risk aversion setting occurred in each sex, but in differing proportions. The key difference was an enhanced presence of high risk aversion settings in females compared to males. This influences excess earnings on average of males (significance and 95% confidence interval for the mean difference Δ*μ*: p = 10^–7^, Δ*μ ϵ* [8.1, 16.9], male mean = 228, sd = 30.4, female mean = 216, sd = 25.25). This represents a small to medium effect size (Cohens d = 0.44), however we note that for this type of study, which is heavily affected by interindividual differences, current literature suggests, that small effect sizes are common and the associated effects potentially relevant (see Funder & Ozer, 2019). We note that the relevant extremes of the risk aversion distribution are very well reproduced in model-derived simulations, as evidenced by the confusion matrix in ***Figure 2A***. Thus, we rule out the possible explanation of this being due to systematic skewing caused through model-fitting. The distribution of membership in one of the 5 earnings quintiles by sex can be seen in ***Figure 2C***. This closely matches the respective risk aversion distribution in our generative model. The effects of risk aversion on the obtained earnings of an investor can be seen in ***Figure 2D***.

**Figure 3:**
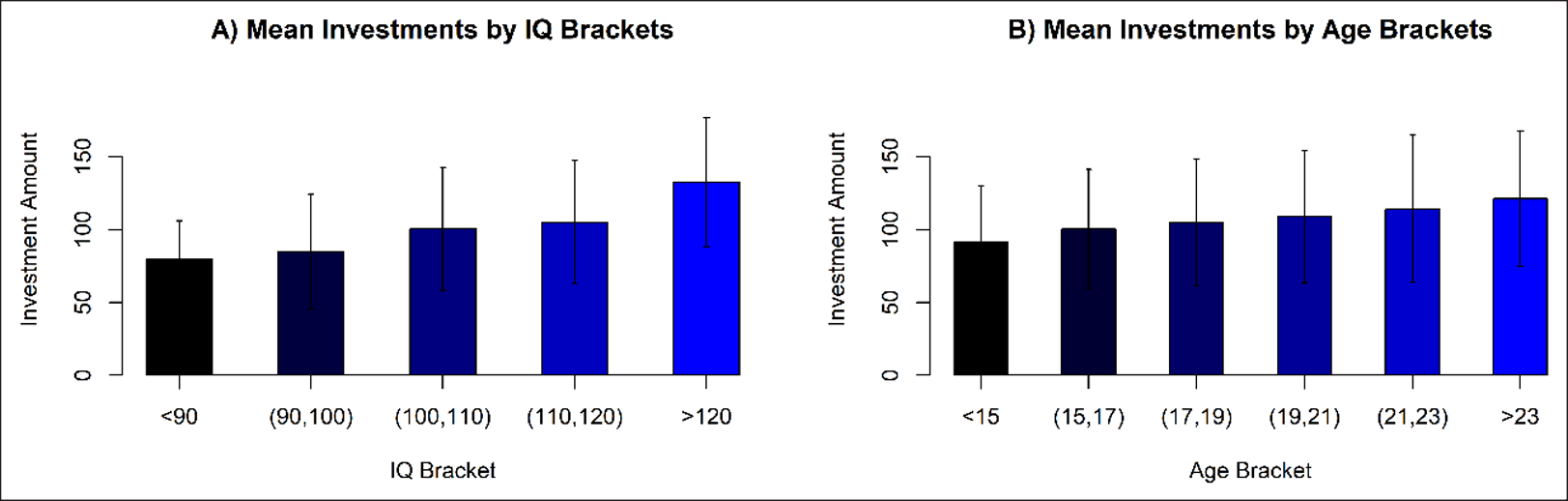
**A)** Average investments separated by raw (non-age-standardized) IQ groups (brackets of width 10 IQ points). Error bars are standard deviations (SD). **B)** Average total Investments ordered by participant age, in 2 year brackets. Error bars are standard deviations (SD).

To illustrate the demographic effects found here, in an empirical correlate of risk aversion (namely the total investments), ***Figure 3A*** and ***3B*** show the relationships between raw (non-age-standardized) IQ vs. Investment and Age vs. Investment. The monotonic effect of each of these variables on total investments is readily observed.

### Relation between inequality aversion and demographic variables

For inequality aversion (*φ = α*), the ultimate model was:


11 \[
l(\mathbb{P}[\alpha < i]) = b_{{\mathrm{0}},i}^{\;\alpha } + b_{Age}^{\;\alpha }Age + b_{SES}^{\;\alpha }SES + \;b_{sex}^{\;\alpha }sex + b_{Ag{e^{\mathrm{2}}}}^{\;\alpha }Ag{e^{\mathrm{2}}} + \epsilon,
\]

We found a significant ( \[
b_{Sex}^\alpha = 0.47
\] , *p* = 0.001) effect of sex on inequality aversion, females having a higher empirical frequency of inequality aversion values above 0. One empirical correlate of this is the percentage of male or female actors making 0-investments in the data set (total number 247 of 0-investments, 159 by male, 88 by female participants) which is 64.4% of 0-investments for male and 35.6% for female participants. Furthermore, we found a marginally significant effect of age on inequality aversion ( \[
b_{Age}^\alpha = - 0.14
\] , *p* = 0.06). The effects of age squared ( \[
b_{Ag{e^2}}^\alpha = 0.11
\] , *p* = 0.16) and SES ( \[
b_{SES}^\alpha = - 0.11
\] , *p* = 0.13) were not significant despite surviving model selection.

### Relation between Irritation Awareness, Irritability, Planning, Theory of Mind and demographic variables

For several parameters (*φ = q*(ζ),ζ,*p,k*), we obtained no noteworthy (i.e. surviving model selection non-constant) relation with the basic demographic variables.

### Relation between Inverse Temperature and demographic variables

For the inverse temperature (*φ = β*), the ultimate model was:


12 \[
\beta = b_{\mathrm{0}}^\beta + b_{IQ}^\beta IQ + b_{I{Q^{\mathrm{2}}}}^\beta I{Q^{\mathrm{2}}} + b_{SE{S^{\mathrm{2}}}}^\beta SE{S^{\mathrm{2}}} + \epsilon,
\]

We found a significant though small effect of raw (non-age-standardized) IQ ( \[
b_{IQ}^\beta = 0.024
\] , *p* = 0.03), a (marginally) significant effect of IQ squared ( \[
b_{I{Q^2}}^\beta = 0.015
\] , *p* = 0.07), and no significant effect of *SES*^2^ ( \[
b_{SE{S^2}}^\beta = - 0.009
\] , *p* = 0.14), despite the latter factor surviving model selection. This suggests that our current model fits slightly better for higher IQ subjects.

### Correlations between model parameters

We then investigated the correlations between model parameters in this human data sample. The correlation coefficients and their p-values can be seen in ***Tables 4*** and ***5*** respectively below:

**Table 4:** Kendall’s tau correlations (below diagonal) of the subject parameters derived from the minimum NLL fit.


	*k*	*P*	ω	β	α	ζ	*Q*(ζ)

***k*, ToM Level**	1						

***P*, Planning**	0.0003	1					

**ω, Risk Aversion**	0.097	0.13	1				

**β, Inverse Temperature**	–0.015	0.01	–0.1	1			

**α, Inequality Aversion**	0.06	0.12	0.34	–0.13	1		

**ζ, Irritability**	0.038	–0.0002	0.05	–0.089	0.04	1	

***q*(ζ), Irritation Awareness**	–0.046	–0.12	0.22	–0.29	0.06	0.007	1


**Table 5:** p-values (below diagonal) of the subject parameters derived from the minimum NLL fit. Asterisk (*) signifies correlations with a p-Value below 0.05. P-values below 10^–13^ denoted as 0.


	*k*	*P*	ω	β	α	ζ	*Q*(ζ)

***k*, ToM Level**	0						

***P*, Planning**	0.99	0					

**ω, Risk Aversion**	0.001*	0.000005*	0				

**β, Inverse Temperature**	0.65	0.7	0.0005*	0			

**α, Inequality Aversion**	0.07	0.00015*	0*	0.00004*	0		

**ζ, Irritability**	0.26	0.99	0.1	0.005*	0.2	0	

***q*(ζ), Irritation Awareness**	0.14	0.000039*	0*	0*	0.049*	0.8	0


Risk Aversion correlates positively with inequality aversion, planning, theory of mind level and irritation awareness, as well as negatively with the inverse temperature parameter. Hence subjects with high risk aversion would tend to prefer equitable outcomes and demonstrate longer planning, while subjects with lower risk aversion would tend to have higher choice certainty. Inequality aversion correlates negatively with inverse temperature and positively with risk aversion, planning and irritation awareness. This suggests that more inequality averse subjects tended to be more cautious when engaging in the exchange. The inverse temperature parameter correlates negatively with risk aversion, irritability, irritation awareness and inequality aversion, suggesting that subjects optimizing their outcome followed more consistent strategies. Irritation awareness correlates negatively with planning and the inverse temperature and positive with risk aversion and inequality aversion, suggesting that subjects more aware of partner retaliation were also more risk averse, yet had a tendency to be slightly more inequality averse.

## Discussion

We analysed the decision-making behaviour of a large cross-sectional sample (n = 784) of 14 to 25 year-olds performing as investors in a social exchange task, in which they chose their preferred level of investment to optimise their own preferences about earnings of themselves and their partners. We employed an interactive-agent-based model that quantified investor behaviour according to seven key characteristics, or parameters. In total, these key characteristics explained almost 70% of the variance of the summed investments, hence giving a close account of an important statistic of the success of the social interaction. We found that the novel model-based measure of risk aversion in the MRT (introduced in Hula et al, 2018) alone accounted for 44% of the variance in the total earnings of the subjects.

The socio-economic risk aversion parameter consistently decreased with age, resulting in on average higher investment amounts and earnings in young adults compared to adolescents. This may be an important mechanistic factor in the observed (but not undisputed) relationship in the literature between trust and age during this developmental period (see van den Bos et al, 2010). Risk aversion was lower on average in participants with higher IQ and males compared to females, controlling for age and socio-economic status. We also found a trend towards inequity averse play to be stronger in females compared to males.

The various dependencies of risk aversion relate in interesting but complex ways to the existing literature of how reward sensitivity and strategies of play develops in adolescence in a multitude of economic games (see an overview in Sutter et al, 2019). This may partly be because our definition of risk in this task is slightly different from that in other framings, for instance including an endowment effect. We observed that socio-economic risk aversion decreased with age between 14 and 25 years of age. Previous findings are that risk aversion outside the context of social exchange increases with age much later in the life span (see Rutledge et al, 2016), or is independent of age (for instance van de Groep et al, 2018). In contrast, and consistent with our findings, trust and reciprocity in trust games has been found to increase over adolescence (in particular see van den Bos et al, 2010, Reniers et al, 2016).

We note that the reported effect sizes could be seen as small, however this is quite typical for the kind of study of interindividual differences, that we performed. A recent discussion of this aspect can be found in Funder & Ozer, 2019.

Our finding that risk aversion decreased with IQ is consistent with observations that IQ is correlated with higher investments and earnings, and trust and reciprocity (see Proto, Rustichini & Sofianos, 2017). Here, we caution that IQ is subject to factors such as motivation (see Breuning & Zella, 1978, Duckworth et al, 2011, Borghans, Meijers & Weel, 2013), education, socio-economic factors (see Baker et al, 2015) and even the life context in which people from different countries and ethnicities grow up. This is consistent with socioeconomic risk-aversion in our task being a motivational parameter, rather than one reflecting how costly a more complex or accurate model of the task might be. The latter might be expected under a simplistic assumption that IQ reflects essential cognitive capacity.

The differences in risk aversion that we found between females and males is consistent with others in the behavioural literature (see van de Groep et al, 2018, van den Akker et al, 2020, Gneezy, Leonard & List, 2009, Charness & Gneezy, 2012) and in recent fMRI studies (see Lemmers-Jansen et al, 2017). However not every study found this effect (see van den Bos et al, 2010).

Our findings on the effects of sex on inequality aversion are also consistent with the literature on adults (see for instance Fehr, Naef & Schmidt, 2006). It might be related to differences in empathy and perspective taking, that were found in studies of adolescent behaviours too (see Van der Graaff, 2014). There may be a sex specific difference in the distribution of response strategies to taking risks and social threat. However, we note that the effect on inequality aversion though significant, is relatively small and would need further investigation.

Finally, it is notable that in our model we observed a novel correlation of risk aversion with socioeconomic status. Risk aversion was higher (and thus trust measured as investments lower) in subjects in more adverse socio-economic conditions (although a quadratic effect in SES counteracts this observation for the high end of the SES spectrum i.e. the most adverse status subjects, in our coding). This is in line with studies finding positive effects of economic support during adolescence having effects on the behaviour in economic games (see Amir, Jordan & Rand, 2018, Luo et al, 2018). This finding is new (and will require replication), yet studies using other measures of trust to measure the effect of SES do exist (see Navarro-Carrillo, Valor-Segura & Moya, 2018, Stamos, Altsitsiadis & Dewitte, 2019). Notably, the study by Amir, Jordan & Rand, 2018 did also find an association between increases in risk aversion and more adverse conditions during childhood, but in that study the subjects with more adverse backgrounds also were more likely to offer higher splits on average in an ultimatum game. The simultaneous presence of a linear and a quadratic effect (with opposing signs) of SES on risk aversion in our analysis suggests a more complicated relationship, on which further research is needed.

Concerning the relation between IQ and the inverse temperature parameter, it appears that higher IQ subjects played less stochastically given how they evaluated the options available, at least according to our model. This is consistent with recent work from our group showing that a measure of decision-making which loads heavily on inverse-temperature across many, mostly non-social, tasks has a similar relation to IQ (see Moutoussis et al, 2021).

Some limitations apply to the model-based results of this study. Overall, the reliability, predictive and construct validity of MRT tasks remain to be better established, although the present study does provide evidence for the external validity of our task. In our case, the model parameters needed to be discrete because of computational limitations. While current work on easing this restriction is ongoing, we are not yet able to let parameters like inequality aversion and risk aversion or inverse temperature vary freely. Secondly, parameter recoverability is always subject to limitations, in particular in a task where partner actions strongly influence the dynamics of the interaction (i.e. responses to cooperative or uncooperative partner reveal different aspects of investment behaviour respectively, but only 1 dyad is available per subject). Changes in task design that may address some of these issues in the future include providing trustees with a base income as well, ensuring that the trustee can always act non-trivially or obtaining multiple games per subject. The validity of ‘risk aversion’ in socio-economic exchanges needs to be characterised both in terms of test-retest but also construct validity, including its relation to other measures of risk preference. Multiple different games and multiple instances of the same game played per subject could potentially yield more robust classifications.

Limitations of the overall study design can also be addressed in future research. The cross-sectional nature of the present sample limited us to statements about population distributions rather than within-subject, developmental effects. Finally, studies that focus specifically on sex or gender and socio-economic status may be necessary to further investigate the mechanisms through which they shape decision making and risk aversion. Specifically, we speculate that the lived experience of young people influences their confidence to take risks in economic exchanges like the present one.

Neurobiologically, changes in trust behaviour have been related to neural results on cortical thickness, which can provide a venue for further research using structural MRI and resting state data (see Bellucci et al, 2018, Tamnes et al, 2018) in combination with multi round trust game data. Furthermore, a previous study in fact relates a lack of increase in neural reward response in adolescent males with depressive symptoms in these subjects (see Morgan et al, 2013), so the relation between risk aversion in the trust game and depressive symptoms warrants further investigation.
